# Do Sex-Related Differences of Comorbidity Burden and/or In-Hospital Mortality Exist in Cancer Patients? A Retrospective Study in an Internal Medicine Setting

**DOI:** 10.3390/life11030261

**Published:** 2021-03-22

**Authors:** Alfredo De Giorgi, Fabio Fabbian, Rosaria Cappadona, Ruana Tiseo, Christian Molino, Elisa Misurati, Edoardo Gambuti, Caterina Savriè, Benedetta Boari, Valeria Raparelli, Roberto Manfredini

**Affiliations:** 1 Clinica Medica Unit, Azienda Ospedaliero-University of Ferrara, 44121 Ferrara, Italy; degiorgialfredo@libero.it (A.D.G.); ruana.tiseo@gmail.com (R.T.); christianmolino@tiscali.it (C.M.); e.misurati@ospfe.it (E.M.); edoardo.gambuti@edu.unife.it (E.G.); caterina.savrie@edu.unife.it (C.S.); brobdt@unife.it (B.B.); 2 Department of Medical Sciences, University of Ferrara, 44121 Ferrara, Italy; rosaria.cappdona@unife.it; 3 Department of Translational Medicine, University of Ferrara, 44121 Ferrara, Italy; valeria.raparelli@unife.it

**Keywords:** in-hospital mortality, comorbidity, cancer, sex-related differences, internal medicine

## Abstract

Cancer represents important comorbidity, and data on outcomes are usually derived from selected oncologic units. Our aim was to evaluate possible sex-related differences and factors associated with in-hospital mortality (IHM) in a consecutive cohort of elderly patients with cancer admitted to internal medicine. We included all patients admitted to our department with a diagnosis of cancer during 2018. Based on the International Classification of Diseases, 9th Revision, Clinical Modification, demography, comorbidity burden, and diagnostic procedures were evaluated, with IHM as our outcome. We evaluated 955 subjects with cancer (23.9% of total hospital admissions), 42.9% were males, and the mean age was 76.4 ± 11.4 years. Metastatic cancer was diagnosed in 18.2%. The deceased group had a higher modified Elixhauser Index (17.6 ± 7.7 vs. 14 ± 7.3, *p* < 0.001), prevalence of cachexia (17.9% vs. 7.2%, *p* < 0.001), and presence of metastasis (27.8% vs. 16.3%, *p* = 0.001) than survivors. Females had a higher age (77.4 ± 11.4 vs. 75.5 ± 11.4, *p* = 0.013), and lower comorbidity (10.2 ± 5.9 vs. 12.0 ± 5.6, *p* < 0.001) than males. IHM was not significantly different among sex groups, but it was independently associated with cachexia and metastasis only in women. Comorbidities are highly prevalent in patients with cancer admitted to the internal medicine setting and are associated with an increased risk of all-cause mortality, especially in female elderly patients with advanced disease.

## 1. Introduction

Along with the progressive aging of the population, the incidence and prevalence of chronic conditions are increasing worldwide, with a rise in long-term conditions and multimorbidity [1,2]. The prevalence of multimorbidity is progressively increasing, and Italian data referring to a decade ago estimated it with ranges from 55 to 98% [3]. In the United States, multiple chronic conditions are diagnosed in about 3 out of 4 subjects aged 65 years and older as well, and often require hospitalization [4].

Cancer also represents an important comorbidity. Data from England and Wales reported that cancer deaths raised from 135,635 to 143,638 between 2006 and 2014, and it has been estimated that people aged over 65 will increase as well as people dying over the age of 85 up to 53%, and cancer deaths will rise up to 208,636 deaths per year in 2040 [5]. On the other hand, comorbidity burden plays a crucial role in cancer patients, and together with age, represent important determinants of survival. In fact, the prevalence of comorbidity in patients with breast, prostate, lung, and colorectal cancer was 32.2%, 30.5%, 52.9%, and 40.7%, respectively [6]. Moreover, sex is a key biological factor affecting the development of many cancer types, and sex-specific differences, either molecular and immunologic, have been reported [7,8]. There are considerable differences between male and female subpopulations in terms of cancer incidence, prognosis, and mortality [9], and, for certain types of cancer, sex may be regarded not only as a prognostic factor but as a predictive factor as well [10]. Since data on comorbidity and cancer are usually drawn by selected populations admitted to selected oncologic units, the aim of this study was to analyze the relationship between in-hospital mortality (IHM) and comorbidity burden, as well as possible sex-related differences, in a consecutive cohort of elderly patients with cancer admitted to a general internal medicine setting.

## 2. Materials and Methods

This retrospective study was run in agreement with the declaration of Helsinki of 1975, revised in 2013. Regional Health authorities deleted from the database available for analysis any subject identifiers, aiming at maintaining data anonymity and confidentiality. Thus, none of the patients could be identified, either in this study or in the entire extracted database. The study was conducted in agreement with the existing Italian disposition-by-law (G.U. n.76, 31 March 2008) and, due to the study design, ethics committee approval was not required.

### 2.1. Patient Selection and Eligibility

This study included all hospitalized patients with cancer in 2018, recorded in the database of the University Hospital St. Anna of Ferrara, Region Emilia-Romagna (RER) of Italy, and maintained by the Center for Health Statistics. The University Hospital St. Anna of Ferrara has been storing in an electronic database all the discharge hospital sheets (DHS) of hospitalizations since 1999. Available data include age, date and department of hospital admission and discharge, vital status at discharge, length of stay, main and up to 6 accessory discharge diagnoses, and the most important diagnostic procedures, based on the International Classification of Diseases, 9th Revision, Clinical Modification (ICD-9-CM). Individual personal data and any other potential identifiers have been removed from the database provided for this study. The St. Anna Hospital (Azienda Ospedaliero-Universitaria) represents the hub center of the province of Ferrara (about 350,000 inhabitants) that have also 3 smaller spoke hospitals. The province of Ferrara had the oldest population of the region Emilia-Romagna: Over-65 people account for 27.7% (vs. 23.8% of the regional average), 14.6% are over 75 years (vs. 12.7% of the regional average), and 4.4% are aged more than 85 years (vs. 4% of the regional average) [11]. The S.Anna Hospital had 660 beds and was provided with all facilities, with the exclusion of cardiothoracic surgery. The yearly patient-flow to the Emergency Department (ED) was approximately 90,000, and 1/3 of all hospital admissions were addressed to the Department of Medicine, provided with 165 total beds, open 7/7 and 24/24 to the ED admissions. 

All patients admitted to the Department of Medicine with a diagnosis of cancer (year 2018), both present on admission or diagnosed during hospitalization, and based on ICD-9-CM, have been included in our study. To evaluate the comorbidity burden, we used a modified Elixhauser Index (mEI), a novel score proposed by our group for patients admitted to internal medicine wards [12]. The score included the following conditions: Age, sex, presence of renal diseases, neurological disorders, lymphoma, solid tumor with metastasis, ischemic heart disease, congestive heart disease, coagulopathy, fluid and electrolyte disorders, liver disease, weight loss, and metastatic cancer. Each condition was related to peculiar points, and the sum of the different points represents the score. As the population selected in this study were patients with cancer, we adjusted the score for the points awarded to cancer or metastasis, i.e., such diseases were not considered in the final score. 

Thus, we indicated as mEI-Ad the applied score, adjusted for hematologic, solid cancer, and metastasis. Points were assigned to each condition following Quan et al. guidelines [13]. IHM risk was considered significant when the score was >40, overcoming the value of 60%. [12]. In-hospital mortality (IHM) and length of hospital stay (LOS) were calculated, with IHM chosen as the main outcome of the study. Moreover, total diagnostic procedures, invasive diagnostic procedures, and blood cell transfusions were also evaluated.

### 2.2. Data Analysis 

A descriptive analysis was performed, and data were expressed as absolute numbers, percentages, and means ± SD. Univariate analysis was performed to define the difference between survivors and deceased subjects and females and male patients. Statistical analysis was conducted using the χ^2^, Student t-tests and Mann–Whitney-U test, as appropriate. Moreover, a logistic regression analysis was carried out in order to assess the independent parameters associated with IHM. In the model comorbidity burden, metastasis, and evidence of cachexia were considered as independent variables. The model was evaluated in the whole population, as well as in the subgroups by sex. Odds ratios (ORs) and their 95% confidence intervals (95% C.I.) were reported. For statistical analysis, SPSS 26.0 for Windows was utilized.

## 3. Results

Subjects with cancer hospitalized in the internal medicine department during the year 2018 were 955 (23.9% of total hospital admissions), 410 (42.9%) males, and 545 (57.1%) females. The mean age was 76.4 ± 11.4 years. Organ solid cancer was evident in 94.2% of cases, whilst subjects with hematologic neoplasm were 6.8% of cases (10 patients had both solid and hematologic neoplastic diseases). Cancer was present on admission in 56.3% of subjects, metastatic cancer in 18.2%, and mean mEI-Ad was 11.2 ± 5.7. Length of hospitalization (LOS) was 13.2 ± 10.9 days, and subjects diagnosed with cachexia were 86 (9%). During admission, the mean total non-invasive diagnostic procedures were 4.6 ± 2.7, mean invasive diagnostic procedures were 0.7 ± 1.2, and 17.8% of individuals underwent blood cell transfusions.

Deceased persons were 162 (17%). Subjects with a negative outcome had higher mEI-Ad (17.6 ± 7.7 vs. 14 ± 7.3, *p* < 0.001, and higher prevalence of cachexia (17.9% vs. 7.2%, *p* < 0.001) and presence of metastasis (27.8% vs. 16.3%, *p* = 0.001) than survivors. Females had higher age (77.4 ± 11.4 vs. 75.5 ± 11.4, *p* = 0.013), and lower mEI-Ad (10.2 ± 5.9 vs. 12.0 ± 5.6, *p* < 0.001) compared with males. IHM was not significantly different among females and males (15.1% and 18.3%, respectively), as well as diagnostic intervention and blood transfusions. Although IHM was significantly associated with mEI-Ad only when the population was considered as a whole, it was independently associated with cachexia and metastasis only in women. Description of the entire population is reported in Table 1 and Table 2; comparison between survivors and deceased patients is reported in Table 3; comparison between female and male patients is reported in Table 4; logistic regression analysis, performed in the whole population and subgroups by sex to evaluate risk factors for IHM, is reported in Table 5.

## 4. Discussion

Advanced cancer is an important challenge for patients, caregivers, and health care professionals, impacting patient’s physical and psychosocial well-being. Hospitalizations represent common events in subjects with cancer, and it is more likely that they are unplanned, and more frequent near the end of life. Measurement of comorbidities is a crucial step for health care professionals in order to optimize and personalize the care of older adults with cancer. Again, a sex-oriented attention is important since comorbidities are an important cause of hospital readmission. Readmissions to hospital after discharge are considered adverse, serious, and costly outcomes, and even though readmissions to internal medicine units are often related to age, they are observed mainly in females [14]. The main findings of this study, performed in the real world of an unselected internal medicine setting, are: Subjects with negative outcome had higher comorbidity index, and higher prevalence of cachexia and presence of metastasis than survivors; females had higher age and lower comorbidity, compared with males, IHM was not significantly different among sex subgroups, but it was independently associated with cachexia and metastasis only in women. There were no differences in the operative approach either between deceased and discharged patients and between sex subgroups, in terms of total and mean non-invasive diagnostic procedures, and blood transfusions.

Prognosis is a crucial point for clinical decision-making in cancer patients [15], and scores based on performance status would seem preferable at the forefront of clinical decision-making regarding prognosis [16]. The use of the Charlson Comorbidity Index, a score validated in internal medicine settings [17], has been shown to improve accuracy [18,19]. We used a modified version of the Elixhauser Index, proposed by our group, validated for patients admitted to internal medicine wards [12,20], and also positively tested as a valid predictor of IHM in other diverse conditions. [21,22], which was integrated for this study with specific adjustments for hematologic, solid cancer, and metastasis (mEI-Ad). We found that mEI-Ad could predict IHM independently from cancer-specific clinical features. In particular, every one-unit increase in the mEI-Ad comorbidity index corresponded to a 5.3% rise in the risk of IHM. Interestingly, this finding is in complete agreement with Williams et al. who studied the association between comorbidity and all-cause mortality using various comorbidity algorithms and found that the risk of death increased by 5% for each unit increase in comorbidity burden [23]. As expected, the presence of metastasis was associated with negative outcomes. In fact, the presence of metastasis has been found to be predictive of poor quality of life [24], and early mortality in non-oncologic registries and non-oncologic wards [25,26].

Data from selected samples of patients have reported sex-specific differences in patients with cancer as far as the late 90s when a Spanish study reported that the incidence of gastric cancer was higher for men, but women showed a better prognosis [27]. Recent data from Sweden found that men with non-small cell lung cancer had a consistently poorer prognosis [28], and women with pulmonary resections for lung cancer had a lower risk of death compared with men [29]. Moreover, the female sex was found to show a protective effect on the development of bone metastases [30]. Reports from our country found better progress for women with colorectal cancer [31], and female sex was an independent predictor of long-term survival for advanced biliary tract cancers [32]. However, in renal transplant recipients, the female gender exhibited a stronger association with IHM as well as solid organ cancers and post-transplant lymphoproliferative disorders [33]. We found that women showed lower comorbidities, and this finding is in agreement with the results of a recent study showing a greater functional impairment in females and more comorbidity in males, but no differences in the prognosis [34].

Treatment or over-treatment of patients of cancer has often been debated between ethics and aggressive medical treatment. A trend toward rising intensity of care and treatments in the last months of life of patients with cancer has been reported [35], and often health care professionals prefer to focus on biochemical interventions rather than end-of-life [36]. Thus, palliative care is offered late in the disease pathway, often limited to the last month of life [37]. Although cancer patients are usually admitted to internal medicine settings, available data from Italian hospitals are very limited. A single-center retrospective study, performed in the region of Tuscany, nearest to Emilia-Romagna in terms of both geographic position and health service organization, analyzed 354 patients (54% females) who consecutively died in an internal medicine unit during a 1-year of observation [38]. The authors observed that, in the last 48 h of life, approximately 2/3 of patients underwent at least one blood assay, 1/3 arterial blood gas analysis, and 1/3 at least one procedure, e.g., X-ray, computerized tomography, magnetic resonance, or endoscopy. During their hospital stay, 9% of patients underwent a blood transfusion, and 28% of these were provided in the last 48 hours of life. Our study found no significant difference in the number of diagnostic and/or invasive procedures performed during the hospital stay in subjects deceased or discharged. Moreover, 17.8% of patients received at least one blood transfusion, but once again, no statistical difference was found between deceased and discharged patients. These data underline the need for a clear definition of the diagnostic and therapeutic plans according to prognosis, especially if a shortage of resources is present.

### Limitations

Our study has several limitations. First, this is a single center retrospective study, analyzing only patients admitted to the internal medicine setting. As our study is based on a hospital registry, we did not include data about individuals followed-up by general practitioners or living in nursing homes. Therefore, our results could not be generalizable to subjects with cancer dying in these settings. Second, observational and retrospective studies based on ICD-9-CM codes are characterized by low sensitivity and specificity. In fact, physicians’ ability in codifying hospital procedures and diagnosis could influence the quality of data since different comorbidity scores were promoted from administrative purpose, addressed to economic rather than research reasons. Third, due to the study design, we analyzed only IHM, excluding the events in patients who eventually died after discharge. Fourth, we could not consider clinical or performance parameters, but only the burden of comorbidity, since ICD-9-CM cannot provide information on disease severity, functional status, or intensity of treatment given. Fifth, comorbidity indexes do not include all possible comorbidities, and they usually include those that precedent a statistical process related to mortality. Last but not least, administrative databases cannot provide information on the marital status of our patients, and this condition could be associated with different outcomes. In fact, being unmarried, divorced, or separated has been associated with the poorest cardiovascular outcomes, as well as with a higher risk of all-cause and cancer mortality in males [39,40]. However, even with these limitations, the present study, based on a significant cohort of patients with advanced cancer, could be considered representative of everyday clinical practice of a general internal medicine setting facing elderly, comorbid, and cancer patients.

## 5. Conclusions

Non-neoplastic-related comorbidities are highly prevalent and are associated with an increased risk of all-cause mortality in elderly patients with advanced cancer. In these patients, an early stratification of prognosis could be useful to provide a more appropriate level of care, reducing unnecessary invasive examinations or procedures and rather warranting timely access to palliative assistance. Thus, utilization of appropriate scores and standardized protocols could help in making difficult decisions. However, many factors can influence the management of end-of-life, e.g., personal subjectivity, skills, and experience of either medical and nursing staff, diagnostic suggestions by specialty consultants, and requests from relatives, caregivers, or patients themselves. Currently, we do not have enough evidence of possible sex-related differences. The study by Stefanelli et al. did not provide data on this aspect [38], and we only found that IHM was not significantly different among females and males, although it was independently associated with cachexia and metastasis only in women. Studies on sex-specific differences in comorbidity, outcome, and possible relevance of marital status are also needed in the delicate topic of end-of-life.

## Figures and Tables

**Table 1:** Characteristics of the sample population of patients with cancer admitted to an internal medicine setting (n = 955).

Age (Years)	76.4 ± 11.4
Female/male (n (%))	410 (42.9)/545 (57.1)
Organ solid cancer (n (%))	900 (94.2)
Hematologic neoplasm (n (%))	65 (6.8%)
Cachexia (n (%))	86 (9%)
Metastatic disease (n (%))	174 (18.2%)
Total diagnostic procedures (n)	4.6 ± 2.7
Invasive diagnostic procedures (n)	0.7 ± 1.2
Blood cells transfusions (n (%))	170 (17.8%)
Length of hospital stay (days)	13.2 ± 10.9
Deceased (n (%))	162 (17%)
mEI-Ad	11.24 ± 5.76

mEI-Ad: Modified Elixhauser Index adjusted for hematologic, solid cancer, and metastasis.

**Table 2:** Type of cancer and distribution by sex and age, and percentages of deceased subjects (NS: Not significant).

		Females (n = 410)	Males (n = 545)	*p*	Deceased (n = 162)
Lung cancer	n = 199	57 (13.9%)	142 (26.1%)	<0.001	41 (25.3%)
	Age	75.6 ± 12.4	74.9 ± 9.9	0.699	
Breast cancer	n = 103	103 (25.1%)	-	-	12 (7.4%)
	Age	77.9 ± 11.6	-	-	
Gastrointestinal cancer	n = 409	172 (42%)	237 (43.5%)	NS	73 (45.1%)
	Age	78 ± 10.4	75.5 ± 11.5	0.024	
Kidney cancer	n = 45	16 (3.9%)	29 (5.3%)	NS	5 (3.1%)
	Age	80.9 ± 9.2	77 ± 12.3	0.266	
Prostatic cancer	n = 62	-	62 (11.4%)	-	12 (7.4%)
	Age	-	79.5 ± 7	-	
Bladder cancer	n = 56	15 (3.7%)	41 (7.5%)	0.012	12 (7.4%)
	Age	81.7 ± 10.2	80.9 ± 75	0.769	
Gynecologic cancer	n = 26	26 (6.3%)	-	-	3 (1.9%)
	Age	76.7 ± 11.1	-	-	
Melanoma	n = 12	1 (0.2%)	11 (2%)	0.015	2 (1.2%)
	Age	85	81.7 ± 8.9	-	
Brain cancer	n = 18	10 (2.4%)	8 (1.5%)	NS	1 (0.6%)
	Age	722 ± 13.7	73.9 ± 10	0.776	

**Table 3:** Comparison of clinical characteristics: Discharged and deceased patients.

	Discharged (n = 793)	Deceased (n = 162)	*p*
Age (years)	76.4 ± 11.6	76.1 ± 10.7	NS
Female (n (%))	348 (43.9%)	62 (38.3%)	NS
Male (n (%))	445 (56.1%)	100 (61.7%)	NS
Organ solid cancer (n (%))	751 (94.7%)	149 (92%)	NS
Hematologic neoplasm (n (%))	51 (6.4%)	14 (8.6%)	NS
Cachexia (n (%))	57 (7.2%)	29 (17.9%)	<0.001
Metastatic disease (n (%))	129 (16.3%)	45 (27.8%)	0.001
Blood cells transfusions (n (%))	136 (17.2%)	34 (21%)	NS
Total diagnostic procedures (n)	4.6 ± 2.6	4.7 ± 2.8	NS
Invasive diagnostic procedures (n)	0.6 ± 1.2	0.7 ± 1.4	NS
Length of hospital stay (days)	13.4 ± 10.3	12.6 ± 13.5	NS
mEI-Ad	14 ± 7.3	17.6 ± 7.7	<0.001

mEI-Ad: Modified Elixhauser Index adjusted for hematologic, solid cancer, and metastasis.

**Table 4:** Comparison of clinical characteristics: Subgroups by sex.

	Females (n = 410)	Males (n = 545)	*p*
Age (years)	77.4 ± 11.4	75.5 ± 11.4	0.013
Organ solid cancer (n (%))	387 (94.4%)	513 (94.1%)	NS
Hematologic neoplasm (n (%))	28 (6.8%)	37 (6.8%)	NS
Cachexia (n (%))	34 (8.3%)	52 (5.2%)	NS
Metastatic disease (n (%))	77 (18.8%)	97 (17.8%)	NS
Blood cells transfusions (n (%))	67 (16.3%)	103 (19.6%)	NS
Total diagnostic procedures (n)	4.6 ± 2.6	4.5 ± 2.6	NS
Invasive diagnostic procedures (n)	0.6 ± 1.3	0.7 ± 1.1	NS
Length of hospital stay (days)	13.6 ± 11.9	12.9 ± 10	NS
mEI-Ad	10.23 ± 5.86	12.01 ± 5.56	<0.001
Deceased (n (%))	62 (15.1)	100 (18.3%)	NS

mEI-Ad: Modified Elixhauser Index adjusted for hematologic, solid cancer and metastasis.

**Table 5:** Risk factors for IHM: Logistic regression analysis * (total population and subgroups by sex)**.**

	Odds Ratio	95% Confidence Intervals	*p*
**Total population**			
mEI-Ad	1.034	1.002–1.067	0.036
Cachexia	2.095	1.226–3.578	0.007
Metastatic disease	1.930	1.292–2.883	0.001
**Women**			
mE-Ad	1.037	0.986–1.091	0.161
Cachexia	4.038	1.729–9.430	0.01
Metastatic disease	2.465	1.310–4.639	0.005
**Men**			
mEI-Ad	1.026	0.986–1.069	0.225
Cachexia	1.385	0.679–2.825	0.370
Metastatic disease	1.691	0.999–2.861	0.05

mEIa-Ad: Modified Elixhauser Index adjusted for hematologic, solid cancer and metastasis; * age was excluded from the model.

## Data Availability

The datasets analyzed are available from the corresponding author on reasonable request.
